# Induced Pluripotent Stem Cells Restore Function in a Human Cell Loss Model of Open-Angle Glaucoma

**DOI:** 10.1002/stem.1885

**Published:** 2015-02-17

**Authors:** Diala W Abu-Hassan, Xinbo Li, Eileen I Ryan, Ted S Acott, Mary J Kelley

**Affiliations:** a Department of Ophthalmology, Casey Eye Institute, Oregon Health & Science UniversityPortland, Oregon, USA; b Department of Biochemistry & Molecular Biology, Oregon Health & Science UniversityPortland, Oregon, USA; c Department of Integrative Biosciences, Oregon Health & Science UniversityPortland, Oregon, USA; d Department of Biochemistry & Physiology, University of JordanAmman, Jordan

**Keywords:** Induced pluripotent stem cells, Autologous stem cell transplantation, Cell transplantation, Experimental models, Somatic stem cells, Stem cell transplantation, Tissue regeneration, Transplantation

## Abstract

Normally, trabecular meshwork (TM) and Schlemm's canal inner wall endothelial cells within the aqueous humor outflow pathway maintain intraocular pressure within a narrow safe range. Elevation in intraocular pressure, because of the loss of homeostatic regulation by these outflow pathway cells, is the primary risk factor for vision loss due to glaucomatous optic neuropathy. A notable feature associated with glaucoma is outflow pathway cell loss. Using controlled cell loss in ex vivo perfused human outflow pathway organ culture, we developed compelling experimental evidence that this level of cell loss compromises intraocular pressure homeostatic function. This function was restored by repopulation of the model with fresh TM cells. We then differentiated induced pluripotent stem cells (iPSCs) and used them to repopulate this cell depletion model. These differentiated cells (TM-like iPSCs) became similar to TM cells in both morphology and expression patterns. When transplanted, they were able to fully restore intraocular pressure homeostatic function. This successful transplantation of TM-like iPSCs establishes the conceptual feasibility of using autologous stem cells to restore intraocular pressure regulatory function in open-angle glaucoma patients, providing a novel alternative treatment option. Stem Cells *2015;33:751–761*

## Introduction

Glaucoma, an optic neuropathy, is the second leading cause of blindness affecting over 70 million people worldwide 1,2. Elevated intraocular pressure (IOP) is the primary risk factor for glaucomatous optic nerve damage and is currently the only treatable factor to ameliorate progression of this damage and the resulting permanent loss of vision 1,3. This is true for all forms of glaucoma, including the most common form, open-angle glaucoma (OAG). In OAG, the angle between the cornea and iris remains open and there is no obvious macroscopic or microscopic obstruction to outflow 3.

Elevated IOP in OAG results from increased resistance to the outflow of aqueous humor and reflects compromised IOP homeostasis. Aqueous inflow is relatively pressure insensitive and the outflow resistance, which can be modulated by outflow pathway cells, actually regulates IOP. IOP homeostasis is a natural process in which sustained pressure changes trigger corrective adjustments in the outflow resistance, thereby maintaining IOP within a narrow safe range 4. This is normally achieved by trabecular meshwork (TM) and Schlemm's canal (SC) inner wall cells within the outflow pathway (Supporting Information Fig. S1A). It is thought that these cells sense sustained IOP changes as mechanical stretching or distortion and adjust the extracellular matrix (ECM) to correct the outflow resistance 4–9. Experimentally, IOP homeostasis is studied in ex vivo perfused anterior segment organ culture 4. Typically, after perfusion in organ culture (Supporting Information Fig. S1B) at normal physiologic pressure, the pressure is doubled to produce a sustained pressure challenge. This triggers the IOP homeostatic response and over several days TM/SC cells reduce the outflow resistance and restore normal IOP 4. This parallels the events in normal eyes in vivo 4. Loss of the IOP homeostatic response is thought to be a hallmark of OAG 4.

Although the molecular etiology of glaucoma remains poorly understood, genetic and some environmental factors are important 7,10–13. No single gene defect is responsible for more than a small fraction of OAG cases. However, reduced TM cellularity is significantly associated with OAG 14. Although no experimental evidence that this TM cell loss is physiologically significant has been presented previously, it has been assumed that this would compromise outflow pathway function, that is, IOP regulation 15. OAG is a disease of aging, with incidence increasing after 40, and the outflow pathway is subjected to numerous cellular insults. The normal outflow pathway exhibits a more modest decline in cellularity with age 14,16,17. Outflow pathway stem cells have been identified, which normally replace lost TM cells 18–20. Presumably, they are unable to keep up with the sustained cell loss associated with OAG 15. Consequently, stem cell therapy to restore lost cellular functions associated with diseases shows particular clinical promise 21–24. Many diseases of aging, including OAG, exhibit selective cell loss 14–16. Although the TM is an immune-privileged site, using autologous stem cells, such as induced pluripotent stem cells (iPSCs) derived from the patient's own fibroblasts, seems optimal. Restoration of compromised tissue function by iPSC replacement therapy is thus of considerable interest 21,23.

Here, we show that controlled outflow pathway cell loss does indeed impair IOP homeostasis, which is the primary function of the outflow pathway 4,8. Replacement of these lost cells with either fresh TM cells or with differentiated iPSCs restores IOP homeostatic function. These studies establish the potential for a regenerative treatment using autologous stem cells to restore the key function to this diseased tissue and thus avoid progressive glaucomatous vision loss.

## Materials and Methods

### Materials

Saponin detergent was from Sigma Life Sciences (St. Louis, MO; http://www.sigmaaldrich.com/life-science.html?) and Live/Dead Viability/Cytotoxicity Kit and fluorescent-labeled zymosan particles for phagocytosis assay were from Molecular Probes/Invitrogen, (Eugene, OR; http://www.lifetechnologies.com/us/en/home/brands/molecular-probes.html). Aqueous humor was collected from fresh postmortem porcine eyes by inserting a 27 gauge needle through the cornea into the anterior chamber and slowly removing 100–150 µl/eye. This was stored at −20°C and centrifuged at 15,000*g* for 10 minutes before use. Antibodies used were: CD44 (352-020, Ancell (Bayport, MN; http://www.ancell.com/) and ab65829, Abcam; Cambridge, UK; http://www.abcam.com/); CHI3L1 (ab88847; Abcam); α3 integrin (NBP1-19724, Novus Biologicals; Littleton, CO; http://www.novusbio.com/); KLF4 (ab72543, Abcam); LAMP1 (ab25630, Abcam); Wnt1 (ab15251, Abcam); AQP1 (sc-20810, Santa Cruz; Santa Cruz, CA; http://www.scbt.com/); NANOG (sc-33759, Santa Cruz); OCT3/4 (sc-5279, Santa Cruz); SOX2 (sc-20088, Santa Cruz); and α-tubulin (04-1117, Millipore; Darmstadt, Germany; http://www.emdmillipore.com).

### TM Cells

Primary TM cells, isolated from porcine and human eyes, were maintained as previously described using TM cell growth medium (medium-glucose Dulbecco's modified Eagle medium [DMEM], a 1:1 mix of high glucose and low glucose media, supplemented with 10% fetal bovine serum [Hyclone/Thermo Scientific; Waltham, MA; http://www.thermoscientific.com/thermo-scientific-hyclone.html?] and 1% antibiotic-antimycotic [100×; Life Technologies; Carlsbad, CA; http://www.lifetechnologies.com)]). Primary TM cells were used from passage 2 to 5 25–28.

### Perfused Anterior Segment Organ Culture

Perfused human and porcine anterior segment organ culture used modifications of methods previously described 29–32. An illustration of the outflow apparatus using constant pressure perfusion is shown in Supporting Information Figure S1B. Human donor eyes were from Lion's Vision Gift, Portland, Oregon. Human donor tissue protocols were approved by the Oregon Health & Science University Institutional Review Board and were conducted in accordance with the tenets of the Declaration of Helsinki. Supporting Information Table S1 contains donor information. Human anterior segments were cultured in stationary organ culture in TM growth medium without serum for 5–7 days to facilitate recovery from postmortem storage 33 before they were mounted in the perfusion apparatus. Porcine anterior segments, obtained within a few hours postmortem, were mounted in the perfusion apparatus immediately. Anterior segments were perfused using a constant 1× pressure (8.34 mmHg) with average flow rates of 1–7 µl/minute for humans and 2–8 µl/minute for porcine as measured gravimetrically. For a sustained 2× pressure challenge to trigger the IOP homeostatic response, the perfusion head was increased to 16.68 mmHg by raising the perfusion reservoir. All perfusions were with TM cell growth medium but without serum. Flow rates were measured by weighing fluid loss from the perfusion reservoir and presented as normalized flow rates normalized to the initial pretreatment baseline flow rate. Outflow facility (*C*) is defined as the flow rate in µl/minute divided by the perfusion pressure in mmHg.

### Live/Dead Assay

The Live/Dead Viability/Cytotoxicity Assay Kit provides a two-color fluorescence cell viability assay in which calcein, acetoxymethyl becomes fluorescent within live cells and nuclear ethidium homodimer (EthD-1) staining indicates cell death. Cells cultured on chamber slides were washed with sterile phosphate buffered saline (PBS) and incubated in Live/Dead solution for 30 minutes at 37°C. Cells were then washed with PBS and visualized by confocal microscopy. Live (green) and dead (red nuclei) cells were counted directly and presented as percentage of the cells that were dead.

Porcine and human anterior segments were similarly soaked in Live/Dead solution, washed with PBS, and viewed en Face or after radial or frontal sections had been cut (see below). For anterior segments, simple cell counts are impractical due to the highly convoluted “trabecular” structure where cells extend in every direction and their borders are difficult to see even in Z-stacks on the confocal microscope. Here, we used counts of EthD-1 stained nuclei as dead cells per field. As a proxy for number of live cells per field, we measured the amount of calcein, acetoxymethyl fluorescence per field. Both measurements were per field volume based on X- and Y-length scales and confocal vertical Z-stack height and were evaluated using ImageJ software (http://imagej.nih.gov/ij/).

### Microscopic Views and Sectioning

As used herein, en Face indicates that anterior segment was viewed directly from the viewpoint of the anterior chamber looking down at the outer TM beams in the direction of aqueous humor flow (arrow in Supporting Information Fig. S1A). Radial sections were cut vertically along a radius of the anterior segment from the center of the cornea to the outer rim of the sclera. This provides an end-on view of the outflow pathway looking into SC, as is shown in the upper portion of Supporting Information Figure S1A. Frontal sections are cut across a pie-shaped wedge of an anterior segment and are perpendicular to radial sections. The cut begins at the outer TM beams from the anterior chamber viewpoint and passes down through the TM, through SC and then out the front of the eye between the cornea and sclera. The frontal cut bisects SC and the cut surface viewed end-on provides a view of an arc of SC with the cornea/sclera below and the TM above 34.

### Saponin Treatment of Cultured TM Cells

Porcine TM cells, grown on glass chamber slides for 48 hours until confluent, were washed with PBS and exposed for 5 minutes to saponin (0.001%, 0.01%, 0.025%, 0.05%, or 0.1%) dissolved in serum-free medium-glucose DMEM. Saponin was removed and cells were rinsed gently with PBS and subjected to Live/Dead staining. Twenty images were captured per treatment condition and live, dead, and total cells were counted in each image.

### Saponin Treatment of Perfused Anterior Segment Organ Cultures

After anterior segments were stabilized at 1× pressure for 24–48 hours in the perfusion system, perfusion fluid was replaced with saponin (0.01%) in serum-free TM growth medium. This was perfused-in and the flow was stopped for 7 or 10 minutes, for human or porcine, respectively. Saponin was then exchanged-out with serum-free TM growth medium and flow was resumed at 1× pressure. Baseline flow was re-established for 24 hours before the pressure head was doubled (2×) by raising the perfusion reservoir to produce a sustained IOP homeostatic pressure challenge.

### Human iPSCs and Control Cells

Human iPSCs (DF6–9-9T.B), derived from dermal foreskin fibroblasts, were from WiCell Laboratories (Madison, WI; http://www.wicell.org/) and were grown and maintained as per the company's instructions. Normal adult human dermal fibroblasts (DF) were from American Type Culture Collection (Manassas, VA; http://www.atcc.org) and cultured according to their instructions. Human umbilical vein endothelial cells (HUVECs) were a gift from Dr. Nabil Alkayed (Anesthesiology and Perioperative Medicine, Oregon Health and Science University, Portland, OR), and were cultured according to the supplier's instructions (Life Technologies).

### Generation of Embryoid Bodies and Differentiation of iPSCs

To generate uniform small size embryoid bodies (EBs) on a large scale, medium sized undifferentiated iPSC colonies were detached from six-well plates by treatment with Accutase (Innovative Cell Technologies: San Diego, CA; http://www.accutase.com/). Cells were transferred to AggreWell plates and incubated in AggreWell medium (Stem Cell Technologies: Vancouver, British Columbia, Canada; http://www.stemcell.com) for 24 hours. The EBs from AggreWell plates were then transferred to six-well plates and differentiation was initiated.

EBs were cultured on TM cell-derived ECM in a mixture of standard TM cell growth medium, AggreWell medium, and TM cell conditioned medium (25%, 50%, and 25%, respectively; which we designated as DiffMedium). The TM-derived ECM was obtained by culturing porcine TM cells for 7 days to produce an extensive ECM. All the TM cells were removed by treatment for 1 hour with 0.1% saponin followed by extensive washing with PBS. ECM was then incubated for several days in PBS before use. No cells could be detected after this time. The conditioned medium was collected after 48 hours from cultured serum-free human TM cells and centrifuged at 15,000*xg* for 15 minutes. The EBs were grown on TM ECM in DiffMedium, which was changed every other day, and maintained in culture for 30 days. After 30 days, the differentiated cells were cultured in 100% TM cell growth medium and passaged 1:3 with trypsin, similar to TM cells, for up to seven passages.

### Western Immunoblotting and Immunohistochemistry

Human TM, iPS, and TM-like iPSCs were grown on six-well plates until confluent. Cell lysates were collected using a RIPA buffer mixed with a protease inhibitor cocktail (Sigma-Aldrich). Protein concentrations were measured using a BCA kit from Pierce Biotechnology (Thermo Scientific; Rockford, IL; http://www.piercenet.com). Loading buffer with 0.1 M dithiothreitol was added to the lysates and samples were boiled for 15 minutes. Equal amounts of protein (20 µg) were loaded per lane in SDS/PAGE gels. Gels were run at 120 V for 90 minutes and wet transferred at 4°C to polyvinylidene fluoride membranes. Non-fat dry milk (5%) was used as a blocking buffer. Primary antibodies were used at 1:1,000 dilution in PBS with 0.05% Tween and incubated at 4°C overnight. Secondary antibodies, both rabbit and mouse, were purchased from Rockland Immunochemicals (Limerick, PA; http://www.rockland-inc.com/), diluted in PBS, and incubated for 1 hour at room temperature.

For immunohistochemistry, human TM, iPS, and TM-like cells were grown on Lab-Tec II CC2-coated glass chamber slides (Nalge Nunc, Inc.; Rochester, NY; http://www.thermoscientific.com/en/about-us/general-landing-page/nalgene-labware.html), until 60%–80% confluent. They were fixed with 4% paraformaldehyde and permeabilized with 0.3% Triton X-100 in PBS. Slides were blocked in 5% normal goat serum in PBS, and then primary antibodies for the cell markers were added at a 1:200 dilution. Both Alexa Fluor 488- and 595-conjugated secondary antibodies (Molecular Probes/Invitrogen) were used at a 1:500 dilution.

### Quantitative RT-PCR

Human TM, iPS, and TM-like cells were grown on six-well plates until confluent. Cells were harvested and total RNA was extracted with Trizol (Life Technologies) using the manufacturer's protocol. Reverse transcription using SuperScript III First Strand Synthesis System (Invitrogen) followed the manufacturer's procedure. Primers for Wnt1, CHI3L1, α3 integrin, AQP1, KLF4, NANOG, SOX2, and OCT3/4 were designed using the integrated DNA Technologies (Coralville, IA) website (http://www.idtdna.com). Primer sequences are listed in Supporting Information Table S2. Ribosomal (18S) RNA was used as a housekeeping gene. The DNA Engine quantitative RT-PCR machine (Bio-Rad; Hercules, CA; http://www.bio-rad.com/) and RT^2^ SYBR Green qPCR master mixes (Qiagen; Vento, Netherlands; http://www.qiagen.com/) were used for real-time PCR according to the manufacturer's protocol. The threshold cycle (*C*_t_) was determined for each sample and used to quantify the relative mRNA levels standardized to the measured iPSC counts. Samples were then run on agarose gels for size verification.

### Phagocytosis Assay for TM-Like iPSCs, iPSCs, and Human TM Cells

Fluorescein-labeled zymosan bioparticles and opsonizing reagent were from Invitrogen /Molecular Probes. TM-like iPS, iPS, and human TM cells were cultured on chamber slides until they were 60% confluent. Zymosan particles were opsonized according to the manufacturer's instructions and then incubated with each cell type for an hour at 37°C. Cells were then fixed in 4% paraformaldehyde, perforated with 0.3% Triton X-100 and stained for LAMP1, a lysosomal marker (1:500 dilution) to establish internalization. Using confocal microscopy, the colocalization of zymosan particles with LAMP1 antibody in Qdot-labeled cells was determined.

### Transplantation of Cells into Saponin-Treated Anterior Segments

All the transplantation experiments were conducted similarly. The cells to be transplanted were harvested by trypsin treatment, rinsed thoroughly, and labeled with 3 µl of QDot nanoparticles (Qtracker 655 Cell Labeling Kit; Life Technologies) by incubating them together for an hour at 37°C. Cells were then washed thoroughly to eliminate residual particles and visualized by confocal microscopy to verify nanoparticle uptake and cell numbers were counted.

Human anterior segments were perfused at 1× pressure for 48 hours to establish baseline flow and then treated with saponin for 7 minutes as detailed earlier to remove approximately 1/3 of the TM cells. Saponin was rinsed out extensively and flow was restarted at 1× pressure (8.34 mmHg) for 24 hours. To test for IOP homeostatic responsiveness with a sustained pressure challenge, perfusion pressure was increased to 2× (16.68 mmHg) for 48 hours for both control and saponin-treated anterior segments. Flow was then stopped, the chamber was inverted, cells were injected, and perfused in at 0.5× pressure for 2 hours. To allow the cells to attach, the flow was then stopped overnight. The flow was then resumed at 1× pressure for 24 hours. With the cells attached to the TM, a second sustained pressure challenge was then initiated to test for recovery of the IOP homeostatic response by increasing perfusion pressure to 2×. The IOP homeostatic capability was assessed over several days and the experiments were terminated.

For all transplantations, 300,000 QDot-labeled cells were added per anterior chamber. This included: primary cultured human TM cells, differentiated TM-like iPSCs, EBs mock-differentiated with 5% aqueous humor, normal DF, and HUVECs.

### Statistical Analysis

One-way ANOVA with Dunnett's multiple testing correction or unpaired *t* tests were used to determine statistical significance.

## Results

### Cellular Loss with Saponin Treatment

Previously, a detailed study demonstrated that eyes from glaucoma patients exhibited significant reduction in cell density within the outflow pathway, when compared with normal aged eyes 14,16,17. To create a relatively uniform and controlled level of cell death in normal aged eyes for our studies, we evaluated a number of possible cell depletion agents. The detergent saponin produced a compromise between effective cell death with minimal ECM and outflow pathway structural disruption. To obtain dosages, saponin was applied to cultured porcine TM cells at different concentrations: 0% (vehicle control), 0.001%, 0.01%, 0.025%, 0.05%, and 0.1%, all in serum-free DMEM (Supporting Information Fig. S2). Saponin at 0.05% killed all the TM cells. When saponin was applied at 0.01%, it produced death in 36% ± 9% of TM cells, whereas 0.001% killed 20% ± 3% of cells. Consequently, 0.01% saponin for 7 minutes was selected for human anterior segments to simulate the approximate level of cell loss detected in glaucomatous eyes 14.

**Figure 1:**
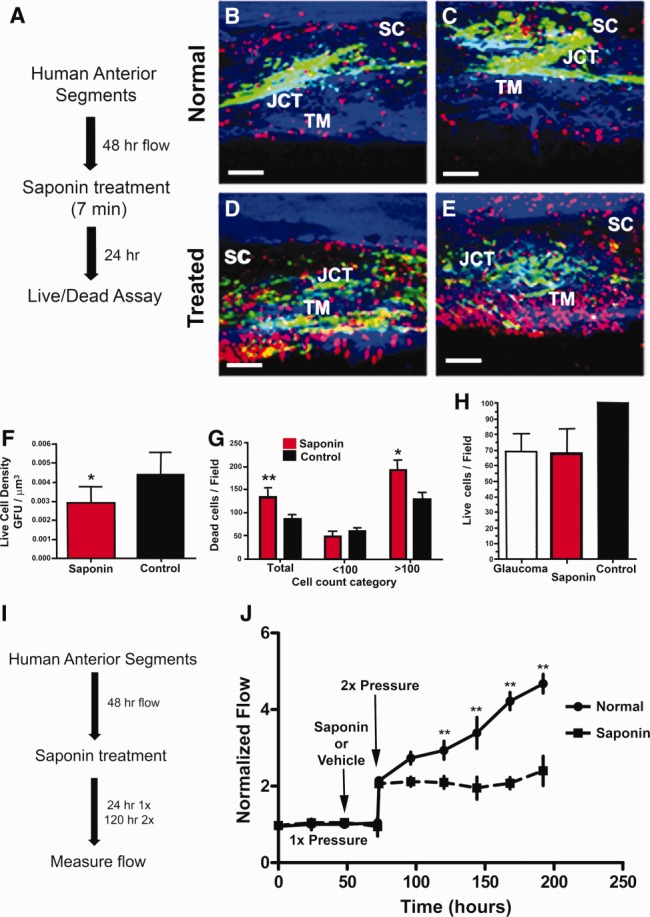
Saponin treatment of human anterior segments. (A): Experimental schematic for cell death assessment after a 7-minute treatment with 0.01% saponin. Frontal sections of normal vehicle-treated control aged anterior segments (B, C) compared to saponin-treated anterior segments (D, E) showing live cells (green) and nuclei of dead cells (red). White scale bars represent 100 µm. (F): Live cell values expressed as GFU per µm field volume, using total area of green fluorescence as a proxy and comparing saponin treated with control. Significance at *p* < .05 is indicated by *. (G): Dead cell count of red nuclei per three-dimensional (3D) field; both total and broken down into fields showing results for less than or more than 100 nuclei. Significance is indicated as **, *p* < .001 and *, *p* < .05. (H): Live cells/field showing comparisons of live cells for glaucoma eyes, saponin-treated, and normal control groups, all normalized to common 3D field volumes. (I): Schematic showing 0.01% saponin treatment pattern for flow studies. (J): Normalized flow rate for perfused human anterior segments treated with saponin or vehicle (normal) and then subjected to intraocular pressure homeostatic 2× pressure challenge. Mean and SEM are shown where *n* = 8 for normal and *n* = 17 for saponin-treated anterior segments with significance determined by one-way ANOVA at *p* < .001 indicated by **. Abbreviations: GFU, green fluorescence units; JCT, juxtacanalicular region; SC, Schlemm's canal; TM, trabecular meshwork.

The most widely accepted experimental model for these studies is perfused anterior segment organ culture (Supporting Information Fig. S1B) 29. Some dead cells were detected in untreated porcine anterior segments, and this was designated basal cell death. However, saponin induced significantly more cell death (Supporting Information Fig. S3). Saponin (0.01%) was injected into the flow line of perfused human anterior segments mounted in the ocular perfusion system (Supporting Information Fig. S1B) and incubated in the TM for 7 minutes before being rinsed out. The live/dead assay was used to determine whether saponin affected the viability of human TM cells and this was viewed in frontal sections of anterior segments (Fig. 1B–1E). The TM has several zones of cells, and saponin was able to penetrate and kill cells in all zones down into the deepest zone, the juxtacanalicular region or JCT (Fig. 1D, 1E), which is the region thought to be responsible for IOP homeostasis 4,8,35. Saponin treatment induced cell death although more live than dead cells still populated the TM, indicating that saponin's effect at this concentration was partial (Fig. 1F, 1G). Saponin-treated anterior segments contained considerably more dead TM cells than vehicle-treated anterior segments (Fig. 1G). Furthermore, comparing the live cells per three-dimensional field in glaucomatous and saponin-treated eyes relative to normal eyes revealed a very similar pattern, that is, 67.42 and 66.42 live cells per field, respectively (Fig. 1H). Thus, this level of saponin treatment induced changes in cell counts that roughly approximate those found in glaucoma.

### Saponin Impairs the IOP Homeostatic Response Which Is Restored by TM Cell Repopulation

To examine the influence of saponin treatment on outflow and on the IOP homeostatic response, we measured the effects of 0.01% saponin treatment on this process (Fig. 1J). Anterior segments were perfused at 1× perfusion pressure until the flow had stabilized. One anterior segment from paired human eyes was then exposed to saponin and the other exposed to vehicle, both for 7 minutes. Saponin or vehicle was then rinsed-out and perfusion was restarted at 1× perfusion pressure. After 24 hours, perfusion pressures were increased to 2× (16.68 mmHg), which serves as a sustained IOP homeostatic pressure challenge 4. Flow rates at 1× were not changed by saponin treatment and they doubled immediately after the pressure was elevated to 2× in both treatment and control anterior segments. However, over several days, the TM cells of the vehicle-treated anterior segments exhibited a typical IOP homeostatic response, that is, they slowly reduced the outflow resistance resulting in a gradual and significant increase in outflow rate (Fig. 1J—solid black line). The saponin-treated anterior segments were not able to produce this IOP homeostatic resistance adjustment, but instead remained at approximately the initial 2× flow rate (Fig. 1J—dashed line). Saponin did not have any direct effect on the outflow resistance, but the consequent cell reduction did degrade the ability of cells to modify the resistance in response to the pressure challenge.

**Figure 2:**
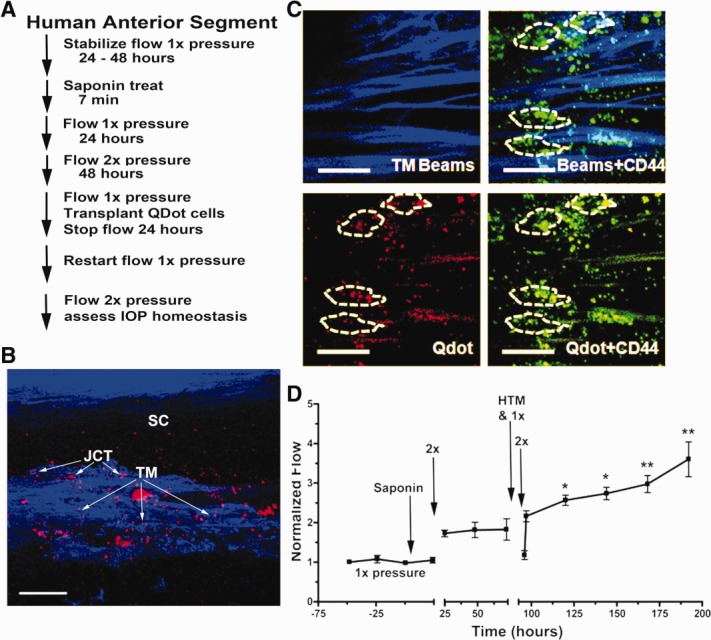
Replacement of saponin-depleted cells with human cultured TM cells. (A): Detailed schematic of treatment protocol. (B): Frontal section showing penetration of QDot (red) labeled HTM cells to all levels of the TM after transplantation. Scale bar is 100 µm. (C): TM beams showing blue autofluorescence from collagen and elastic fibers (TM Beams), CD44 immunohistochemistry (green) to label cell surfaces, and QDot (red) labeled transplanted TM cells. White dashes enclose individual cells, which contain QDots indicating that they were transplanted. Dashes were drawn based on Z-stack three-dimensional scans to identify individual QDot-labeled cells. The scale bar is 100 µm. (D): Transplanted replacement HTM cells (added at the time indicated) restored the intraocular pressure homeostatic response to 2× pressure elevation, which had been compromised by saponin treatment. Line shows mean for six experiments using separate anterior segments and error bars represent the SEM with significance by one-way ANOVA where *, *p* < .05 and **, *p* < .001. Abbreviations: HTM, human trabecular meshwork; JCT, juxtacanalicular region; SC, Schlemm's canal; TM, trabecular meshwork.

Although anterior segments exposed to saponin lost the ability to adjust the outflow resistance when subjected to a 2× pressure challenge, this capability was regained when cultured human TM cells were added back and allowed to attach and integrate into the anterior segments (Fig. 2A–2C). After saponin-treatment and assessment of response to a 2× pressure challenge, human TM cells that had been labeled with red fluorescent QDots for identification purposes were injected into the perfusion line, allowed to flow into the TM at 0.5× pressure for 2 hours, and then the pressure was reduced to 0 mmHg overnight to facilitate cell attachment. Perfusion pressure was then reinitiated, maintained at 1× to re-establish the baseline flow, then increased to 2×, and the flow was measured for several days to assess capability to exhibit an IOP homeostatic response (Fig. 2D). When examined by confocal microscopy (Fig. 2B, 2C), QDot-labeled transplanted TM cells were observed throughout the TM. The transplanted cells appeared to have attached to the TM beams and to inner layers of TM including the JCT (Fig. 2B, 2C). Although the IOP homeostatic response to 2× pressure was lost after saponin treatment, it was regained after the TM cells were added back and allowed to integrate into the open areas (Fig. 2D).

### iPSC EBs Differentiate to be Similar to Human TM Cells

**Figure 3:**
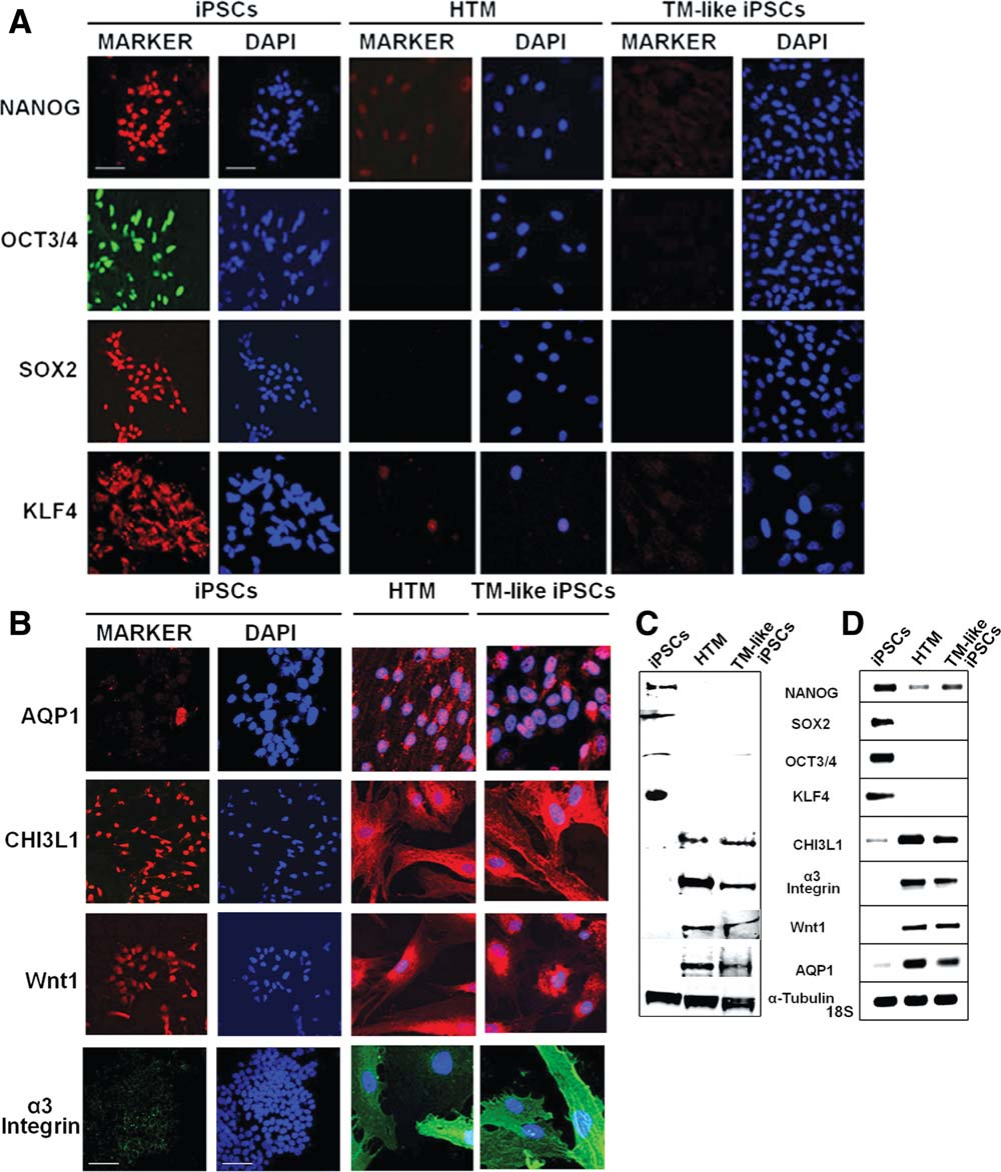
Biomarker expression comparison between iPSCs, TM, and differentiated TM-like iPSCs. (A): Immunohistochemical comparison of levels of four stem cell markers, NANOG, OCT3/4, SOX2, and KLF4 by these three cell types. Cell nuclei are stained with DAPI (blue). All panels are exactly the same size with scale bar = 100 µm. (B): TM cell markers as expressed by iPSCs, HTM, and TM-like iPSC. DAPI nuclear stain is blue, all panels are the exact same size and scales are identical with the white scale bars = 100 µm. (C): Western immunoblot showing levels of these two groups of proteins and (d) gels from quantitative RT-PCR analysis showing levels of mRNA expression for these genes. Loading controls are α-tubulin for Western immunoblots and 18S ribosomal subunit for mRNA gels. Abbreviations: HTM, human trabecular meshwork; iPSCs, induced pluripotent stem cells; TM, trabecular meshwork.

To use the iPSCs, they were differentiated to embryoid bodies, and then to a TM-like cell. Since patient-specific TM cells could only be obtained by an invasive patient surgery, autologous skin iPSCs would be a much preferred source of replacement cells 15. After extensive evaluation of possible methods to differentiate iPSCs (manuscript in preparation), we found conditions that seemed usable. The EBs developed from iPSCs became differentiated or more “TM-like” in appearance after sustained exposure to ECM and conditioned medium, both produced by cultured primary human TM cells. Other treatment combinations were less effective. These TM-like iPSCs resembled human TM cells morphologically (Supporting Information Fig. S4), which have a relatively distinctive appearance 28. Expression of typical stem cell markers, NANOG, OCT3/4, SOX2, and KLF4, 22,23,35 was much higher for iPSCs and negligible for differentiated TM-like iPSCs or human TM cells (Fig. 3A, 3C, 3D). Although there is no specific cell marker for TM cells, CHI3L1, Wnt1, α3 integrin, and AQP1 are typically expressed by TM cells 36–41 and are not expressed or are only lightly expressed by iPSCs (Fig. 3B–3D). The TM-like iPSC expression pattern resembled that of human TM cells much more closely than that of iPSCs (Fig. 3A–3D).

### Phagocytic Capabilities of Differentiated iPSCs

**Figure 4:**
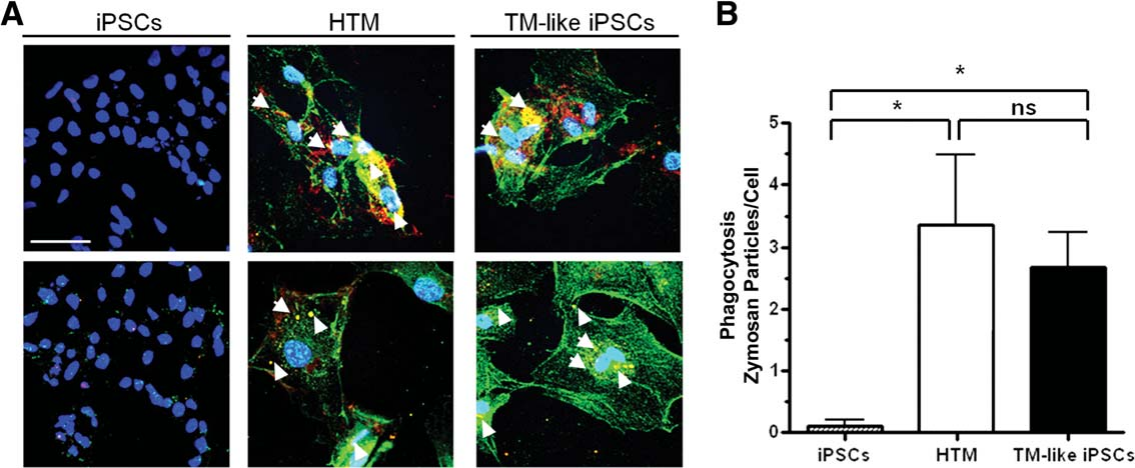
Differentiated TM-like iPSCs can perform phagocytosis, a typical TM cell property. (A): iPSCs, HTM, and differentiated TM-like iPSCs cultured on chamber slides were incubated with fluorescent-labeled zymosan particles for an hour. They were then washed, fixed, and immunostained with LAMP1, a lysosomal marker, to verify internalization. The colocalization (yellow; examples marked with white arrowheads) of zymosan particles (green) with LAMP1 (red) indicates the phagocytosis of the particles. Both HTM and TM-like iPSCs phagocytosed the particles but iPSCs did not. Nuclei were demarcated by DAPI staining (blue). Scale bar represents 100 µm. (B): The number of zymosan particles that colocalize with LAMP1 was counted and the total number was divided by total number of cells in the field. Significance is indicated by * where *p* < .05 and ns indicates not significant. Abbreviations: HTM, human trabecular meshwork; iPSCs, induced pluripotent stem cells; TM, trabecular meshwork.

The outer portion of the TM acts as a filter to remove cells and debris from aqueous humor and is therefore very active in phagocytosis 42,43. However, iPSCs are not effective at phagocytosis. Hence, we examined whether or not the differentiated TM-like iPSCs had acquired phagocytic capability. We exposed iPS, TM, and differentiated TM-like iPSCs to fluorescent-labeled zymosan particles (red) and immunostained afterward for the lysosomal marker LAMP1 (green), to verify that particles were actually internalized and not just absorbed to the cell surface. The results showed that many of the zymosan particles colocalized with LAMP1, indicating internalization, for both human TM and TM-like iPSCs, but not for iPSCs, which exhibit essentially no lysosomes or zymosan uptake (Fig. 4).

### Transplanted TM-Like iPSCs Integrated into the TM and Restored IOP Homeostasis

**Figure 5:**
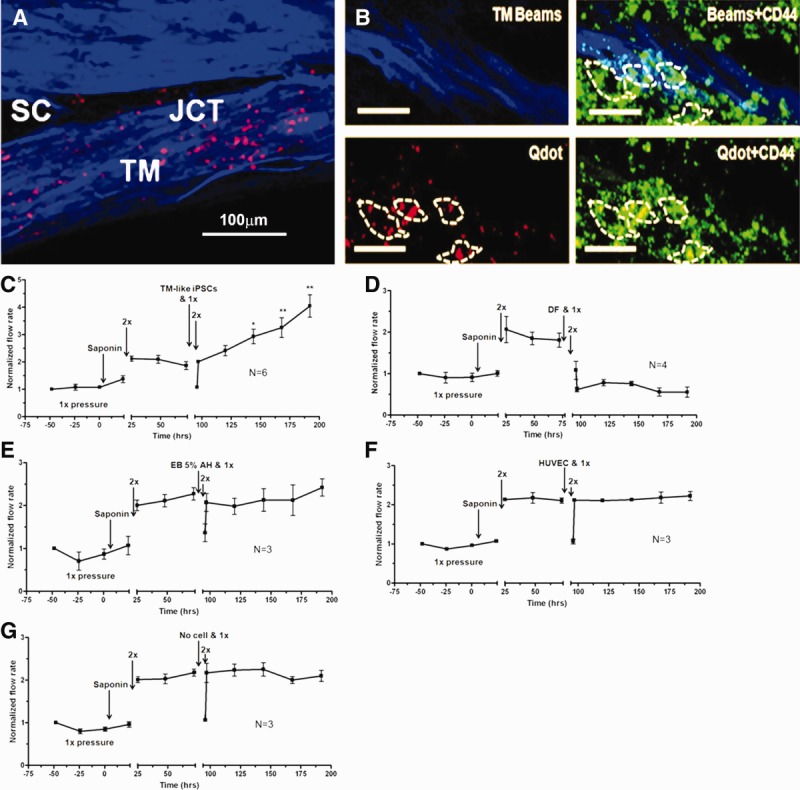
Replacement of saponin-depleted cells with TM-like iPSCs in anterior segments. (A): Confocal analysis of frontal section showing transplanted QDot (red) labeled TM-like iPSCs at all levels of the outflow pathway. Blue shows TM beam collagen and elastic fiber autofluorescence; scale bars are 100 µm. (B): Frontal section after perfusion protocol shows TM beams (blue), cell surface CD44 immunostaining (green), and QDot-labeled transplanted TM-like iPSCs (red). Scale bar indicates 100 µm. White dashes outline individual transplanted cells attached to TM beams. Outlines were determined by scanning through the three-dimensional confocal Z-stacks. (C): After 1× perfusion, saponin was added as indicated, rinsed out, and perfusion resumed for 24 hours at 1× pressure. The 2× pressure challenge gave no intraocular pressure (IOP) homeostatic response. TM-like iPSCs were added and allowed to attach for 24 hours; flow was resumed at 1× pressure and then increased to 2× pressure. A typical IOP homeostatic response now occurred over several days. *n* = 6 experiments with separate anterior segments and significance by one-way ANOVA is *, *p* < .05 and **, *p* < .001. (D): Similar experiment with 300,000 DF transplanted as a control. They actually triggered a reduction in outflow but no IOP response to 2× pressure challenge. (E): Effects of mock-differentiated iPSC EB, which had been exposed only to 5% aqueous humor during a parallel differentiation period, (F) HUVECs, or (G) no cells added at all were compared. Only differentiated TM-like iPSCs produced an IOP homeostatic pressure response. Abbreviations: DF, dermal fibroblasts; EB, embryoid bodies; HTM, human trabecular meshwork; HUVEC, human umbilical vein endothelial cell; iPSCs, induced pluripotent stem cells; TM, trabecular meshwork.

Differentiated TM-like iPSCs were transplanted into saponin-treated human anterior segments (Fig. 5A–5C). The saponin treatment, transplantation process, and pressure challenges were the same as conducted earlier for cultured TM cells (Fig. 2) and detailed in Materials and Methods and outlined in Figure 2A. Many of the 300,000 QDot-labeled differentiated TM-like iPSCs attached and became integrated into the TM. Frontal sections (Fig. 5A, 5B) show that the transplanted cells were found throughout the TM, including the deepest JCT layer, which is thought to be the location of the outflow resistance 8,44. The dashed lines around the Qdot-containing transplanted cells (Fig. 5B) were drawn based on carefully scanning through the confocal Z-stacks to define the CD44-labeled cell surfaces.

After saponin treatment, the 2× pressure challenge did not evoke an IOP homeostatic resistance adjustment, but after the differentiated TM-like iPSCs were transplanted, a second 2× pressure challenge did result in a robust resistance adjustment (Fig. 5C). Similar studies were conducted with transplantation of a selection of several types of control cells (Fig. 5D–5F) or where no cells were added (Fig. 5G). These control cells included DF (Fig. 5D), which are the cell type originally used to develop the iPSCs. Here, the second pressure challenge did not produce an outflow resistance reduction, but actually the DF addition reduced outflow directly. Another control cell, EBs that had been exposed to 5% aqueous humor alone in a parallel control differentiation process did not restore the IOP homeostatic resistance adjustment (Fig. 5E). Addition of HUVECs, which are an endothelial cell as are TM cells (Fig. 5F), or the sham addition (no cells at all) (Fig. 5G) also failed to restore the IOP homeostatic response.

## Discussion

It has long been suspected that the loss of outflow pathway cells observed in OAG would exacerbate the effects of other causal factors associated with this disease 14–17. Here, we show the first experimental evidence that outflow pathway cell depletion in the approximate range observed in glaucoma does indeed compromise the function of the outflow pathway, which is maintaining IOP homeostasis 4,8. Partial repopulation of the cell-depleted tissue with cultured TM cells is able to restore this key outflow pathway function, thereby further strengthening this evidence. In and of itself, this is a major development in understanding the etiology of glaucoma and, perhaps more importantly, understanding why most people do not develop glaucoma even at advanced ages 1,2. Although TM cell loss occurs in normal aging 16,17, that level of loss is not sufficient to curtail IOP homeostatic function 1,3,4,7,8.

Glaucoma is therapeutically a difficult disease in several respects. Current drug therapies all rely on reducing aqueous humor inflow or in diverting outflow through the alternative or uveoscleral outflow pathway 3. In both cases, since a key function of aqueous humor is to bathe and nourish the avascular lens, cornea, and TM, reduced aqueous humor circulation through the conventional TM outflow pathway will further exacerbate the condition of the diseased tissue 1,3,7. In addition, therapeutic compliance remains a very serious issue with published estimates of 27.8%, 50%, or even 75% noncompliance 45–48. This is primarily due to side effects and the requirement for daily self-application of eye drops. Furthermore, these drug treatments often become ineffective, forcing laser treatments or invasive surgical interventions. Although much of glaucoma is of genetic origin, it has become clear that no single genetic locus is responsible for more than a small portion of glaucoma 10–13,49. To date, these various genetic causes of glaucoma have not converged on a single etiologic pathway. Thus, genetic or gene therapy-based resolutions to glaucoma will likely need to be developed individually for many different genetic problems.

These issues and the association of outflow pathway cell loss with glaucoma caused by many genetic or even environmental causes 14 provide the conceptual basis for developing a stem cell therapy for OAG 15. In examining the IOP homeostatic response to cell transplantation, only added TM cells and differentiated TM-like iPSCs were able to restore the IOP homeostatic response in anterior segments partially depleted of TM cells. Control cell transplantations using DF, embryoid bodies that had been mock-differentiated, HUVECs, or mock transplantation (no cells added) were all ineffectual in restoring IOP homeostasis.

Our demonstration that TM cell reduction eliminates the IOP homeostatic response and that TM cell replacement restores this central outflow pathway function provides a strong impetus to develop this new treatment paradigm. Although the outflow pathway resides within an immune-privileged region and is relatively insensitive to normal immune rejection phenomenon, this privilege is clearly not absolute 50,51. Hence, using autologous stem cells seems particularly advantageous. Stem cells, such as iPSCs, which can be developed from patient-specific skin fibroblasts, are thus ideal for this study 15,22–24,35.

## Summary

We demonstrate herein that reduction of outflow pathway cell density compromises IOP homeostasis and that repopulation with TM cells restores this key function. More importantly, iPSCs can be differentiated to resemble TM cells in many key ways and they can restore the primary TM cell function, maintaining IOP homeostasis. This is thus a major conceptual step toward developing a stem cell therapy for OAG.
