# Supplementary figures and images for: Induced Pluripotent Stem Cells Restore Function in a Human Cell Loss Model of Open-Angle Glaucoma
Source: Stem Cells. 2015 Feb 17;33(3):751–61. doi: 10.1002/stem.1885 (PMC4359625; doi:10.1002/stem.1885)

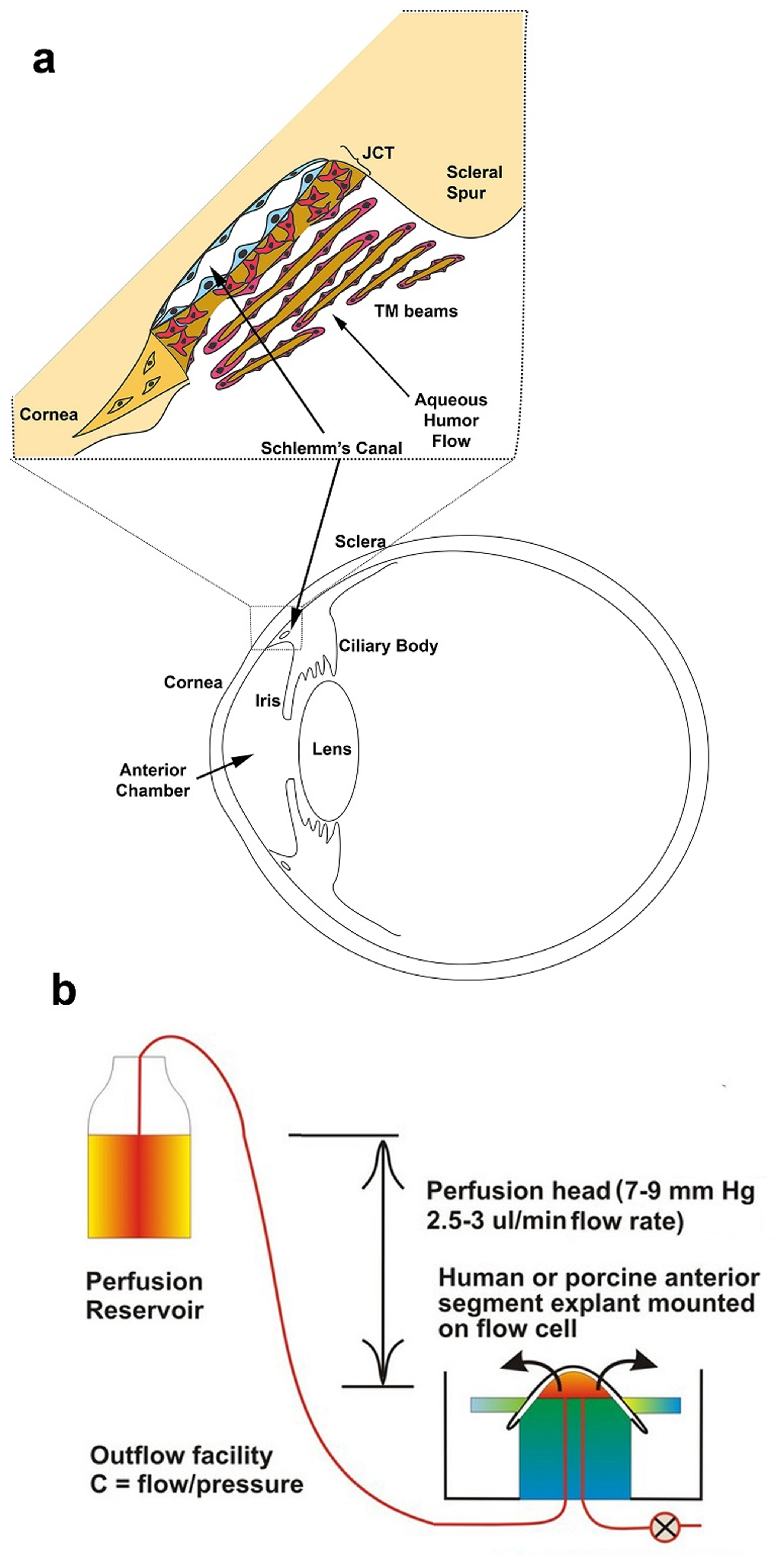

Supplement: Supplementary file 1 [file stem0033-0751-sd1.tif]

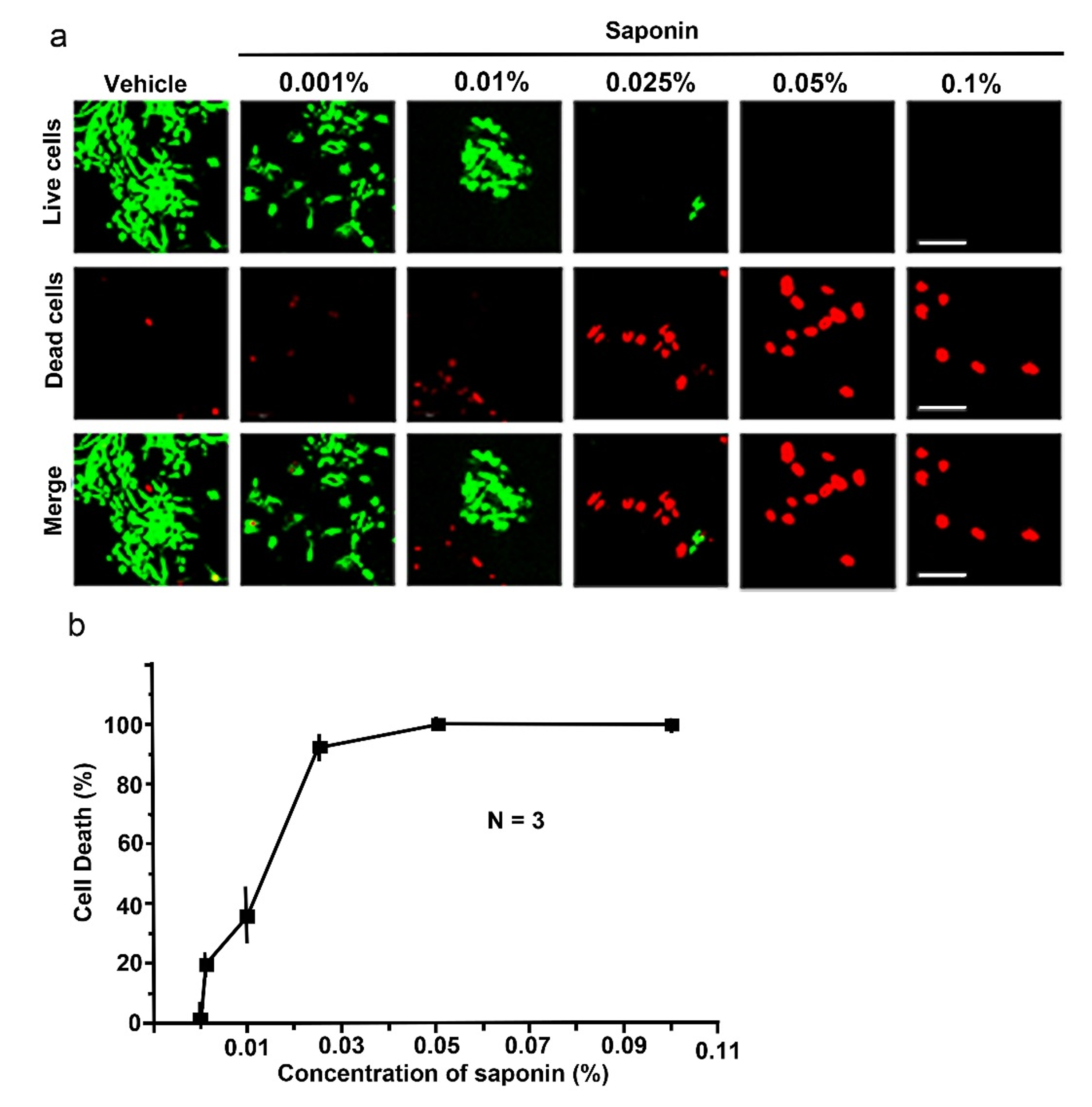

Supplement: Supplementary file 2 [file stem0033-0751-sd2.tif]

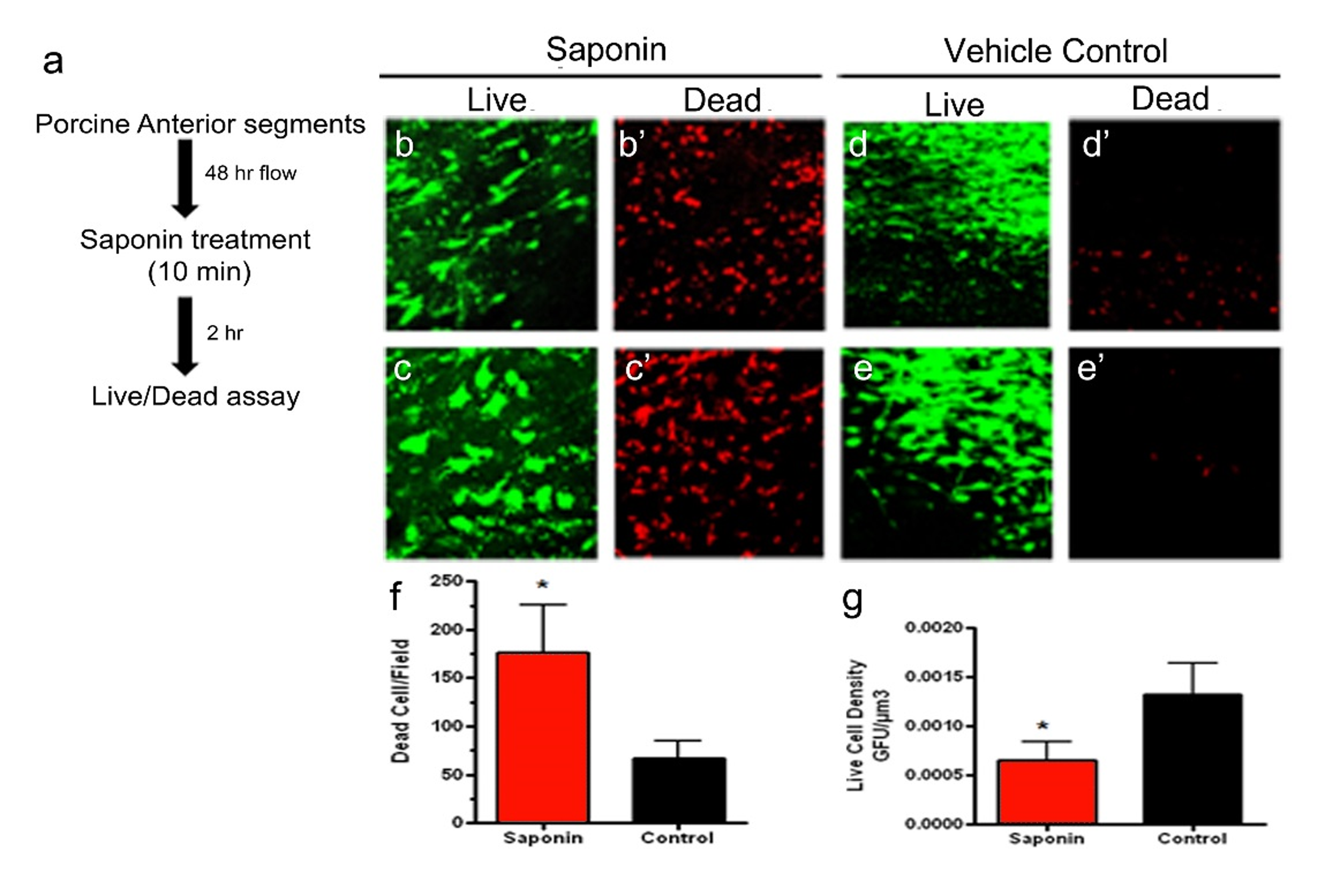

Supplement: Supplementary file 3 [file stem0033-0751-sd3.tif]

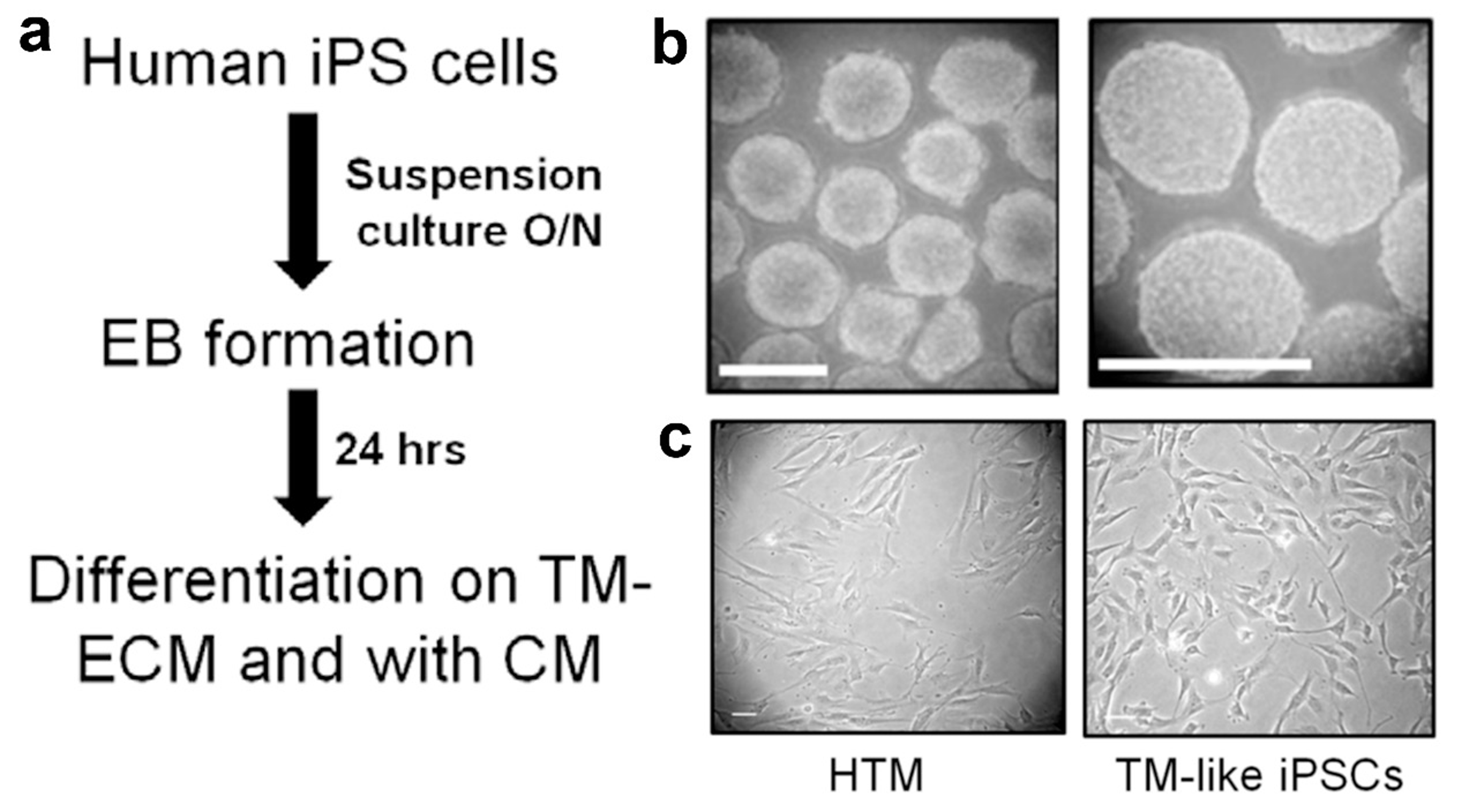

Supplement: Supplementary file 4 [file stem0033-0751-sd4.tif]
