# Supplementary material for: Induced Pluripotent Stem Cells Restore Function in a Human Cell Loss Model of Open-Angle Glaucoma
Source: Stem Cells. 2015 Feb 17;33(3):751–61. doi: 10.1002/stem.1885 (PMC4359625; doi:10.1002/stem.1885)
Supplement: Supplementary file 5 [file stem0033-0751-sd5.doc]

**Supplementary Figure 1.** **Outflow pathway and perfused anterior segment organ culture model.** **a**) Inset shows the human aqueous humor outflow pathway. Aqueous humor flows from the ciliary body through the pupil and out through the trabecular meshwork into Schlemm’s canal where it enters the venous drainage system. The TM outer beams are highly phagocytic. Aqueous humor percolates between the TM beams and then passes through the juxtacanalicular (JCT) region of the TM and across Schlemm’s canal inner wall endothelium (1-5).

Aqueous humor bathes the avascular cornea, lens and TM. Aqueous humor, formed by the ciliary body, flows at a relatively pressure-insensitive rate of around 2.75 μl/min. IOP is thus regulated by adjustments in the resistance to outflow that resides in the deepest portion of the JCT and Schlemm’s canal inner wall. (This diagram is modified significantly from an earlier review (1)).

**b**) Anterior segment organ culture perfusion system (6, 7). Anterior segments, including the cornea, TM and approximately 5 mm of sclera but without the iris, ciliary body or lens, are clamped into a polycarbonate flow cell (6). Culture media is perfused through ports in the bottom of the flow cell, driven by a defined pressure head selected to mimic the physiologic IOP minus the episcleral venous pressure, which is missing in the model. Fluid flow rates are measured gravimetrically. The system is maintained in a standard CO_2_ culture incubator at 37^o^C and 100% humidity.

**Supplementary Figure 2.** **Saponin dose-response curve for TM cell death.** Increasing concentrations of saponin were incubated with cultured porcine TM cells for 10 minutes to obtain a range estimate of cell death using Live/Dead stain (Invitrogen/Molecular Probes). **a**) Live cells are green due to calcein AM uptake, while dead cell nuclei are red due to Ethidium dimer (EthD-1) uptake. Scale bar shows 100 μm. **b**) Red nuclei/field were counted and percentage of dead cells to total cells per field calculated. Error bars show standard error of the mean for 3 separate experiments.

**Supplementary Figure 3**. **Porcine anterior segments treated with saponin at 0.01% or vehicle for 10 minutes.** Two fields of view each for live/dead staining are shown (green and red respectively) in **b** & **b’** or **c** & **c’** compared to parallel vehicle treatment (**d** & **d’** or **e** & **e’**). Dead cells per field were counted and compared in (**f**), while live cells were quantified as total amount of green fluorescence per field in (**g**). Means with standard errors and significance determined by student’s t-test are shown where * indicates P < 0.05.

**Supplementary Figure 4.** **Embryoid bodies (EBs) and differentiated TM-like IPS cells.** **a)** A schematic of the process is shown. **b**) EBs are shown at two magnifications where scale bars are 50 μm. **c)** After differentiation of iPSC EBs using a combination of TM cell produced extracellular matrix and conditioned medium to drive the process, the differentiated TM-like iPSCs exhibit morphology that resembles normal human TM cells (HTM).
