# Supplementary material for: Induced Pluripotent Stem Cells Restore Function in a Human Cell Loss Model of Open-Angle Glaucoma
Source: Stem Cells. 2015 Feb 17;33(3):751–61. doi: 10.1002/stem.1885 (PMC4359625; doi:10.1002/stem.1885)
Supplement: Supplementary file 6 [file stem0033-0751-sd6.docx]

**Supplementary Table 1. Human donor eye information.**

| **Eye Identifier** | **Age** | **Post-Mortem Times** | **Brief Ocular History** |
| --- | --- | --- | --- |
| 2009-0885 OD | 76 | 48 hrs | No remarkable history |
| 2009-0885 OS | 76 | 48 hrs | No remarkable history |
| 2009-0903 OD | 91 | 24 hrs | Pseudophakic |
| 2009-0903 OS | 91 | 24 hrs | No remarkable history |
| 2009-0930 OD | No data available | 48 hrs | No data available |
| 2009-0930 OS | No data available | 48 hrs | No data available |
| 2009-0478 OD | 65 | 48 hrs | No remarkable history |
| 2009-0478 OS | 65 | 48 hrs | No remarkable history |
| 2009-0497 OD | 62 | 24 hrs | No remarkable history |
| 2009-0497 OS | 62 | 24 hrs | No remarkable history |
| 2009-1702 OD | 67 | 48 hrs | No remarkable history |
| 2009-1702 OS | 67 | 48 hrs | No remarkable history |
| 2009-1100 OD | 76 | 58 hrs | Pseudophakic, Possible early macular degeneration |
| 2009-1109 OD | 78 | 40 hrs | No remarkable history |
| 2009-0623 OS | 81 | 48 hrs | No data available |
| 2013-1451 OD |  |  | No data available |
| 2013-1451 OS |  |  | No data available |
| 2013-90 OD | 81 | 48 hrs | Pseudophakic |
| 2013-90 OS | 81 | 48 hrs | Pseudophakic |
| 2013-92 OD | 86 | 48 hrs | Pseudophakic |
| 2013-92 OS | 86 | 48 hrs | Pseudophakic |
| 2013-93 OD | 78 | 48 hrs | No remarkable history |
| 2013-93 OS | 78 | 48 hrs | Pseudophakic |
| 2013-339 OD | 80 | 48 hrs | Pseudophakic |
| 2013-339 OS | 80 | 48 hrs | Pseudophakic |
| 2013-343 OD | 72 | 48 hrs | No remarkable history |
| 2013-343 OS | 72 | 48 hrs | No remarkable history |
| 2013-345 OD | 75 | 48 hrs | No remarkable history |
| 2013-345 OS | 75 | 48 hrs | No remarkable history |
| 2013-490 OD | 77 | 48 hrs | Pseudophakic |
| 2013-490 OS | 77 | 48 hrs | Pseudophakic |
| 2013-492 OD | 86 | 48 hrs | Torn retina, pseudophakic |
| 2013-492 OS | 86 | 48 hrs | No remarkable history |
| 2013-979 OD | No data available | No data available | No data available |
| 2013-994-OS | 84 | 48 hrs | Pseudophakic |
| 2013-1033 OD | 89 | 48 hrs | Pseudophakic, Lasik S/P |
| 2013-1033 OS | 89 | 48 hrs | Pseudophakic, Lasik S/P |
| 2013-1115 OD | 82 | 48 hrs | No remarkable history |
| 2013-1115 OS | 82 | 48 hrs | Lasik, cataract sx, pseudophakic |
| 2013-1079 OD | No data available | No data available | No data available |
| 2013-1079 OS | No data available | No data available | No data available |
| 2013-1110 OD | No data available | No data available | No data available |
| 2013-1110-OS | No data available | No data available | No data available |
| 2013-1243 OD | 90 | 48 hrs | Pseudophakic |
| 2013-1243 OS | 90 | 48 hrs | Pseudophakic |
| 2013-1242 OD | 73 | 48 hrs | Pseudophakic |
| 2013-1251 OD | 91 | 48 hrs | Pseudophakic |
| 2013-1248 OS | 93 | 48 hrs | Blind OS, detached retina, failed scleral buckle, aphakic |
| 2013-489 OD | 86 | 48 hrs | Macular degeneration, cataracts, possible glaucoma |
| 2013-489 OS | 86 | 48 hrs | Macular degeneration, cataracts, possible glaucoma |
