# Supplementary material for: Induced Pluripotent Stem Cells Restore Function in a Human Cell Loss Model of Open-Angle Glaucoma
Source: Stem Cells. 2015 Feb 17;33(3):751–61. doi: 10.1002/stem.1885 (PMC4359625; doi:10.1002/stem.1885)
Supplement: Supplementary file 7 [file stem0033-0751-sd7.docx]

**Supplementary Table 2. Primer Sequences Used in Quantitative RT-PCR**

| **Gene Name** | **DNA Sequence** |  |
| --- | --- | --- |
| AQP1 | Forward: CTCCTGGCTATTGACTACACTG  Reverse: GAAGTCGTAGATGAGTACAGCC | |
| CHI3L1 | Forward: TGTCCAAGCAGTCAAGAGAAG  Reverse: CACATGAGAATTCCTGGGAGAG | |
| Integrin α3 | Forward: CATGGAGAGAAGCTGGGACTG  Reverse: GGTTGTAAGCAAAGCACAGC | |
| KLF4 | Forward: ACCTACACAAAGAGTTCCCATC  Reverse: TGTGTTTACGGTAGTGCCTG | |
| NANOG | Forward: GAAATACCTCAGCCTCCAGC  Reverse: GCGTCACACCATTGCTATTC | |
| OCT3/4 | Forward: GTGAAGCTGGAGAAGGAGAAG  Reverse: TGGTTCGCTTTCTCTTTCGG | |
| SOX2 | Forward: TCTTCGCCTGATTTTCCTCG  Reverse: GTTCTCCTGGGCCATCTTG | |
| Wnt1 | Forward: CTGGCTGGGTTTCTGCTACGC  Reverse: TATCAGACGCCGCTGTTTGCGGC | |
| 18S Ribosomal RNA | Forward: TGTCCTCATGCCTTGGTGCT  Reverse: AGAGATCAAGGGACTTGCTT | |
|  |  | |
