# Supplementary material for: Induced Pluripotent Stem Cells Restore Function in a Human Cell Loss Model of Open-Angle Glaucoma
Source: Stem Cells. 2015 Feb 17;33(3):751–61. doi: 10.1002/stem.1885 (PMC4359625; doi:10.1002/stem.1885)
Supplement: Supplementary file 8 [file stem0033-0751-sd8.docx]

**IPS cells restore function to cell loss glaucoma model**

Diala W Abu-Hassan, Xinbo Li, Eileen I Ryan, Ted S Acott, Mary J Kelley

**SUPPLEMENTARY REFERENCES**

1. Acott TS, and Kelley MJ. Extracellular matrix in the trabecular meshwork (Review). *Exp Eye Res.* 2008;86(543-61.

2. Hogan MJ, Alvarado JA, and Weddell JE. *Histology of the human eye. An atlas and textbook.* Philadelphia: W. B. Saunders Company; 1971.

3. Morrison JC, and Acott TS. In: Morrison JC, and Pollack IP eds. *Glaucoma: Science and Practice*. New York: Thieme; 2003:34-41.

4. Stamer WD, and Acott TS. Current understanding of conventional outflow dysfunction in glaucoma. *Curr Opin Ophthalmol.* 2012;23(2):135-43.

5. Johnson M. 'What controls aqueous humour outflow resistance?'. *Exp Eye Res.* 2006;82(4):545-57.

6. Johnson DH, and Tschumper RC. Human trabecular meshwork organ culture. *Investigative Ophthalmology & Visual Science.* 1987;28(945-53.

7. Johnson DH, and Tschumper RC. The effect of organ culture on human trabecular meshwork. *Experimental Eye Research.* 1989;49(113-27.
